# Development of flow cytometry assays for measuring cell-membrane enzyme activity on individual cells

**DOI:** 10.7150/jca.30813

**Published:** 2020-01-01

**Authors:** Michael Gorry, Toshie Yoneyama, Lazar Vujanovic, Marcia L. Moss, Michelle A. Garlin, Miles A Miller, James Herman, Laura P. Stabile, Nikola L. Vujanovic

**Affiliations:** 1 University of Pittsburgh Cancer Institute, Pittsburgh, PA;; 2 Department of Pathology, University of Pittsburgh;; 3 Department of Immunology, University of Pittsburgh;; 4 Department of Otolaryngology, University of Pittsburgh;; 5 Department of Medicine, University of Pittsburgh;; 6 Department of Pharmacology and Chemical Biology, University of Pittsburgh, Pittsburgh, PA;; 7 VAPHS, Pittsburgh, PA;; 8 BioZyme Inc, Apex, NC;; 9 Center for Systems Biology, Massachusetts General Hospital and Harvard Medical School, Boston, MA.

**Keywords:** Single cell, Cell membrane, Flow cytometry, Enzyme activity, Assays, ADAM10.

## Abstract

**Background:** Cell-membrane expressing enzymes such as ADAM (a disintegrin and metalloproteinase) superfamily members are thought to be key catalysts of vital cellular functions. To directly measure these enzymes and determine their association with particular cells and functions, individual-cell membrane-bound enzyme activity assays are required, but unavailable.

**Methods:** We developed two such assays, using a fluorescence resonance energy transfer (FRET) peptide substrate (FPS) and flow cytometry. One assay measured live-cell natural processing of FPS and binding of its fluorescent product onto individual-cell membrane-bound enzymes. The other assay measured processing of specifically-bound and glutaraldehyde-crosslinked FPS, and consequent generation of its coupled fluorescent product onto individual-cell membrane-bound enzymes.

**Results:** Confocal-microscopy imaging indicated that proteolytic processing of FPS selectively occurred on and labeled cell membrane of individual cells. The new assays measured specific increases of cell-associated FPS fluorescent product in substrate-concentration-, temperature- and time-dependent manners. A large proportion of processed FPS fluorescent products remained cell-associated after cell washing, indicating their binding to cell-membrane expressing enzymes. The assays measured higher levels of cell-associated FPS fluorescent product on wild-type than ADAM10-knockout mouse fibroblasts and on human monocytes than lymphocytes, which correlated with ADAM10 presence and expression levels on cell membrane, respectively. Furthermore, the enzyme activity assays could be combined with fluorescent anti-ADAM10 antibody staining to co-label and more directly associate enzyme activity and ADAM10 protein levels on cell membrane of individual cells.

**Conclusions:** We report on two novel assays for measuring cell-membrane anchored enzyme activity on individual cells, and their potential use to directly study specific biology of cell-surface-expressing proteases.

## Introduction

Cellular enzymes are diverse molecules that selectively convert various molecules (substrates) to functional forms, catalyze main biochemical reactions, and mediate vital cellular functions 1-15. Due to their diverse substrate specificity, different enzymes and their clusters define different cell types, and mediate different cellular functions such as signaling, metabolic processes, differentiation, proliferation, communication and death. 1-15. Among cellular enzymes, those expressed on the cell surface such as ADAM superfamily members have particular roles in cellular biology, as they hydrolyze proximal to the cell surface, shed and activate ectodomains of transmembrane molecules that mediate cell growth, intercellular communication, cell migration, inflammation and cancer pathogenesis 1-5.

To directly investigate the relationships between particular enzymes and diverse cell types and cellular functions, single cell enzyme activity assays are required. Several intracellular single cell enzyme activity assays have been recently developed using improved fluorogenic enzyme substrates 15. The assays include image-based fluorescent microscopy, scanning electrochemical microscopy, capillary electrophoresis and flow cytometry assays. Among them, flow cytometry, being multi-parametric, high throughput, high content, high efficiency and high precision, provides the most compelling information.

While the intracellular single-cell enzyme activity assays have been developed and successfully utilized in a number of studies 15-20, the cell-membrane assays are lacking. We developed and described herein two flow cytometry assays that measure cell-membrane associated enzyme activity at the single cell level. The assays are based on the newly revealed features of the FRET PEPDAB005 substrate that its natural processing by live-cell membrane expressing enzymes generates a fluorescent substrate product which distinctly labels individual cells; and that the substrate sequential specific binding and mild glutaraldehyde crosslinking to cell-membrane anchored enzymes enable unaffected enzyme activity, substrate processing and generation of an enzyme-coupled fluorescent substrate product that labels individual cells. In both assays, a fluorescent substrate product is generated on and labels individual cells depending on their cell-membrane enzyme activity levels, which can be precisely quantified either alone or in combination with specific immuno-staining of particular enzymes and cell markers.

## Materials and Methods

### Reagents

The ADAM10 and ADAM17 moderately-specific FRET enzyme substrate PEPDAB005 (green-color fluorescence with optimal excitation and emission wavelengths of 485 nm and 530 nm, respectively) 21, 22 was obtained from BioZyme Inc (Apex, NC). Cell-membrane selective lipophilic fluorophore DiD (near-infrared fluorescence with optimal excitation and emission of 644 nm and 665 nm, respectively), was purchased from ThermoFisher Scientific (Pittsburgh, PA) as a Vybrant cell-labeling solution. Cell-nuclear DNA specific Hoechst 33342 fluorescent dye (blue fluorescence with optimal excitation and emission of 350 nm and 461 nm, respectively) was purchased from ThermoFisher Scientific. Phycoerythrin (PE)-conjugated mouse monoclonal antibodies (mAb) to human ADAM10 and ADAM17 and corresponding isotype control mAb (red-color fluorescence with excitation and emission wavelengths of 564 nm and 573 nm, respectively) were obtained from R&D Systems (Minneapolis, MN). Paraformaldehyde (PFA) (10% aqueous solution) and Glutaraldehyde (GAL) (2.5% solution in 0.1 M Millonig's Sodium Phosphate Buffer, pH 7.2) were purchased from Electron Microscopy Sciences (Hatfield, PA). RPMI-1640 and DMEM cell-culture media, fetal-calf serum (FCS), Trypsin and enzyme-free cell-dissociation solution were obtained from GIBCO-Life Technologies (Grand Island, NY).

### Cell lines and peripheral blood cells

Human H441 non-small cell lung carcinoma (NSCLC) and K562 myeloid leukemia cell lines were obtained from ATCC (Manassas, VA). They were cultured in RPMI-1640 supplemented with 10% FCS (GIBCO-Life Technologies). Immortalized wild-type (ADAM10^+/-^, clone 37) and ADAM10 knockout (ADAM10^-/-^, clone 8T2) mouse embryonic fibroblasts (MEFs) were provided by Dr. Carl Blobel (Weill Medical College, Cornell University, New York, NY) 23. MEFs were cultured in DMEM supplemented with 10% FCS. Non-adherent K562 cells were passaged twice a week. The adherent cell lines were passaged after reaching 70% confluency using trypsinization. The cell cultures were cyclically restarted from frozen stocks after eight consecutive passages. For experimental use, single-cell suspensions of the adherent cell lines were prepared with the help of the non-enzymatic cell-dissociation solution (GIBCO-Life Technologies) according to company's protocol. After detachment, cells were washed twice in RPMI-1640 or DMEM, to restore divalent cations that are essential for the activity of metalloproteinases. Human peripheral blood mononuclear leukocytes (PBL) were obtained using Phycoll-Hypaque (Sigma-Aldrich, St. Louis, Mo) density gradient centrifugation of healthy donor heparinized peripheral blood provided by the University of Pittsburgh Cancer Institute (UPCI) Lung Cancer (LC) SPORE Tissue Bank (TB) (IRB protocol No. REN16070229/IRB9502100).

### Staining of cell surface-expressing enzymes with PEPDAB005 substrate

Two methods were developed and used to stain enzymes on cell surface: 1) live cell/natural substrate-processing assay (live cell assay), which is based on simple enzymatic reaction-inducing incubation of viable cells in the presence of PEPDAB005 substrate; and 2) fixed cell/crosslinked substrate-enzyme complex processing assay (fixed cell assay), which is based on sequential incubation of viable cells in the presence of PEPDAB005 substrate, cell washing, mild fixation with GAL low concentration and enzymatic reaction-inducing incubation. In both assays, media, reagents and cell suspensions were initially kept on ice during the setup of experiments. In the live cell assay, cell suspensions were prepared in either RPMI-1640 or DMEM supplemented with 0.1% BSA (10^6^ cells/mL) and distributed (10^5^ cells/100 μL) into 5 mL Falcon polypropylene round-bottom tubes (ThermoFisher Scientific). Control cells were suspended in medium alone or supplemented with 10 μM PEPDAB005 solution, and immediately fixed with 100 μL of 2% PFA. Experimental cells were suspended in 10 μM or various concentrations of PEPDAB005 solution and incubated for 30 min or for various time periods at 0^o^C, 21^o^C or 37^o^C. Following these incubations, the experimental cell specimens were fixed with 100 μL of 2 % PFA while being gently mixed. The fixed cell assay is based on the known initiation of enzyme-substrate specific interaction that includes binding by fitting complementary shapes of enzyme(s) and substrate, alignments of atoms in the enzyme active site cleft, non-covalent reactions by electrostatic forces, Van der Waals' forces, hydrogen bonding and hydrophobic interactions, and strong covalent interactions 24. Additionally, mild GAL fixation can cause rapid and irreversible crosslinking of a preformed cell-surface substrate-enzyme complex while preserving intact enzymatic activity and substrate processing in the crosslinked complex. Moreover, GAL fixation crosslinks and immobilizes other cell surface and intracellular proteins, stops all cellular and molecular movements and functions, and preserves morphologically intact cellular membranes and organelles. Cell suspensions were prepared in ice-cold Hank's balanced salt solution (10^5^ cells/100 μL) and incubated in the absence or presence of 5 μM or various concentrations of PEPDAB005 solution for 60 min at 0^o^C (specific binding of substrate to reactive plasma membrane enzymes), washed 3 times with ice-cold Hank's buffer, fixed with 0.005% or various concentrations of GAL for 30 min, incubated for additional 30 min or various time periods at 0^o^C, 21^o^C or 37^o^C, and post-fixed with 2% PFA. The fixed cell assay is expected to be advantageous to and more specific than the live cell assay as it lacks the perpetual enzyme-substrate association/dissociation process, mobility of enzyme-substrate complex, enzyme trafficking and intracellular non-specific processing, all of which may be detrimental to live cell assays.

### Staining of cell surface enzymes with fluorochrome-conjugated antibodies

Standard single color cell surface staining with PE-conjugated isotype-control or anti-ADAM10 or anti-ADAM17 mAb (R&D Systems) was performed as previously described 22, 25.

### Two-color staining of ADAM10 protein and plasma membrane enzymatic activity

Two methods were applied. One method was based on the live cell assay, while the other method on the fixed cell assay. For the first method, H441 cells (10^6^/mL) were suspended in RPMI-1640 medium supplemented with 0.1% BSA, distributed (10^5^ cells/100 μL) into 5-mL round bottom polypropylene tubes (Falcon), mixed with 10 μg of PE-conjugated isotype control or anti-human ADAM10 mAb (R&D Systems), incubated for 30 min on ice and washed twice with the same medium used for preparing cell suspension. After washing, cells were either suspended in the medium alone or 10 μM PEPDAB005 solutions, incubated for 30 min at 21^o^C, and fixed with 2% PFA. For the second method, K562 cells (10^6^/mL) were suspended in Hank's buffer, distributed (10^5^ cells/100 μL) into 5-mL round bottom polypropylene tubes (Falcon), mixed with 10 μg of PE-conjugated IgG control antibody, anti-human ADAM10 antibody and/or 5 μM PEPDAB005 solution, incubated at 0^o^C for 60 min, washed three times with Hank's buffer, fixed with 0.005% GAL at 0^o^C for 30 min, incubated at 21^o^C for 30 min and post-fixed with 2% PFA.

### Flow cytometry analysis

Single-color and two-color flow cytometry analyses were performed on a BD Accuri™ C6 cytometer (Beckman Coulter, Brea, CA), as previously described 22, 25. Data were analyzed using FlowJo v10 (FlowJo, LLC; Ashland, OR).

### Confocal microscopy

Plasma membrane of viable H441 cells were specifically labeled with Vybrant DiD solution (ThermoFisher Scientific) diluted in RPMI-1640 by their co-incubation at 21^o^C for 5 min. These cells were then washed in RPMI-1640 medium, exposed to 10 μM PEPDAB005 substrate solution in RPMI-1640 supplemented with 0.1% BSA at 21^o^C for 30 min, washed and fixed with 2% PFA solution. During fixation, cells were treated with 0.1% BSA, 10 μg/mL Hoechst 33342, and 0.01% sodium Azide on ice, rinsed, and immediately imaged using the Olympus FV1000 confocal laser scanning microscopy system with a XLUMPLFLN 60x water immersion objective (NA 1.0; Olympus America). Images were scanned sequentially using 405 nm, 473 nm, and 635 nm diode lasers in combination with BA430-455 nm, BA490-540 nm and BA655-755 nm emission filters (Olympus), respectively. *Z*-plane resolution sections were obtained using 1 μm thick optical cutting, and imaged. Fluorescence co-localization of PEPDAB005 and DiD was quantified by measuring fluorescence intensity along line of optical section profiles that extended radially across the DiD-labeled cell surface using ImageJ. Data were background-subtracted based on autofluorescence detected in samples not exposed to PEPDAB005 but otherwise processed and imaged under identical conditions (negative control samples), and signals were normalized to the maximum average fluorescence along the radial profile.

## Results

### Cell-mediated processing of PEPDAB005 substrate generates a fluorescent product that labels cell membrane of individual cells

**Figure 1:**
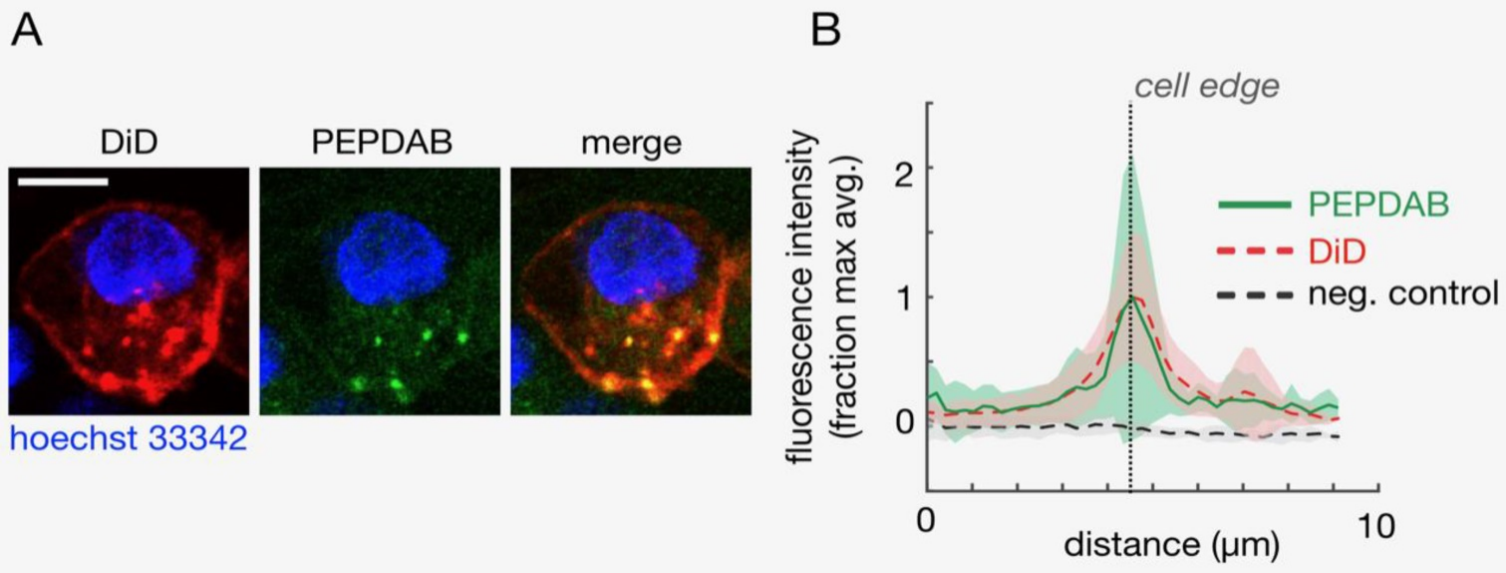
** Cells process PEPDAB005 substrate generating a fluorescent product that binds to and labels cell membrane of individual cells.** Viable H441 cells were sequentially stained with the lipophilic dye DiD by specific labelling of cell membrane, cell-membrane enzyme processed PEPDAB005 substrate and nuclear-DNA specific dye Hoechst 33342, and imaged using confocal microscopy. *Z*-plane resolution sections were obtained using 1 μm thick optical cutting of cells. **(A)** Images of a representative *Z*-plane section of an H441 cell are shown. Scale bar is 10 μm. **(B)** Corresponding to data as in **A**, profiles of fluorescence intensity measured radially across the DiD-labeled cell surface of *Z*-plane section images are presented. Thick lines and shading denote mean +/- standard deviation, respectively (n=10).

Plasma membrane-expressing enzymes such as ADAM superfamily members have catalytic domains oriented outward and function on the cell surface. Several of these enzymes, including ADAM10 and ADAM17, mediate vital cellular functions 1-5. H441 NSCLC and K562 leukemia cells express on cell membrane high ADAM10 and low ADAM17 levels (22 Supplementary Fig. 1), and ADAM10 sheddase activity (ADAM10sa) is a dominant metalloproteinase (MP) activity of NSCLC and likely leukemia cells 22, 26. Therefore, as PEPDAB005 substrate is moderately specific for and similarly susceptible to ADAM10sa and ADAM17sa, it could serve as a surrogate marker of cell-membrane associated ADAM10sa of H441 and K562 cells 22, 26. We wanted to know whether PEPDAB005 substrate could label individual cells by being selectively processed by and generating a bound fluorescent product to cell-membrane expressing enzymes. First, we visualized this hypothetical process using confocal microscopy. Viable H441 cells were sequentially stained with the near-infrared fluorescence lipophilic dye DiD, specifically labelling plasma membrane, the cell-enzyme processed PEPDAB005 substrate green fluorescence product, labeling enzyme activity, and the blue fluorescence dye Hoechst 33342, specifically labeling cell nuclei. Using the confocal microscopy, these three-color stained cells were optically sliced into 1 μm thick *Z*-plane resolution sections, and the cell sections were imaged and analyzed. The green fluorescence of the processed PEPDAB005 substrate product and the red fluorescence of DiD specific staining of cell membrane were consistently co-localized (Figs. 1A, 1B), as shown in the “merge” image by the orange staining (co-localization of lower-level green fluorescence and higher-level red fluorescence) and especially by the multiple well-defined yellow-stained micro patches (co-localization of high-level green fluorescence and high-level red fluorescence) (Fig. 1A); as well as by the automated measurement showing the overlapping of the red and green fluorescence in multiple optical cell sections (Fig. 1B). The majority of co-localized staining was found on the cell surface (edge) as an almost continuous layer of orange staining intercalated with several yellow micro-patches. However, a minor amount of co-localized green and red fluorescence was also found in the cytoplasm as a few oval orange- and yellow-stained micro-structures. These findings demonstrated that PEPDAB005 substrate was selectively processed by cell-membrane anchored enzymes generating *in situ* a fluorescent substrate product that labeled cell membrane of individual cells. The presence of co-localized staining in the cytoplasm indicated that, under the utilized experimental conditions, a few small parts of the cell membrane containing membrane-anchored enzyme/substrate-product complex were endocytosed forming endosomes.

**Figure 2:**
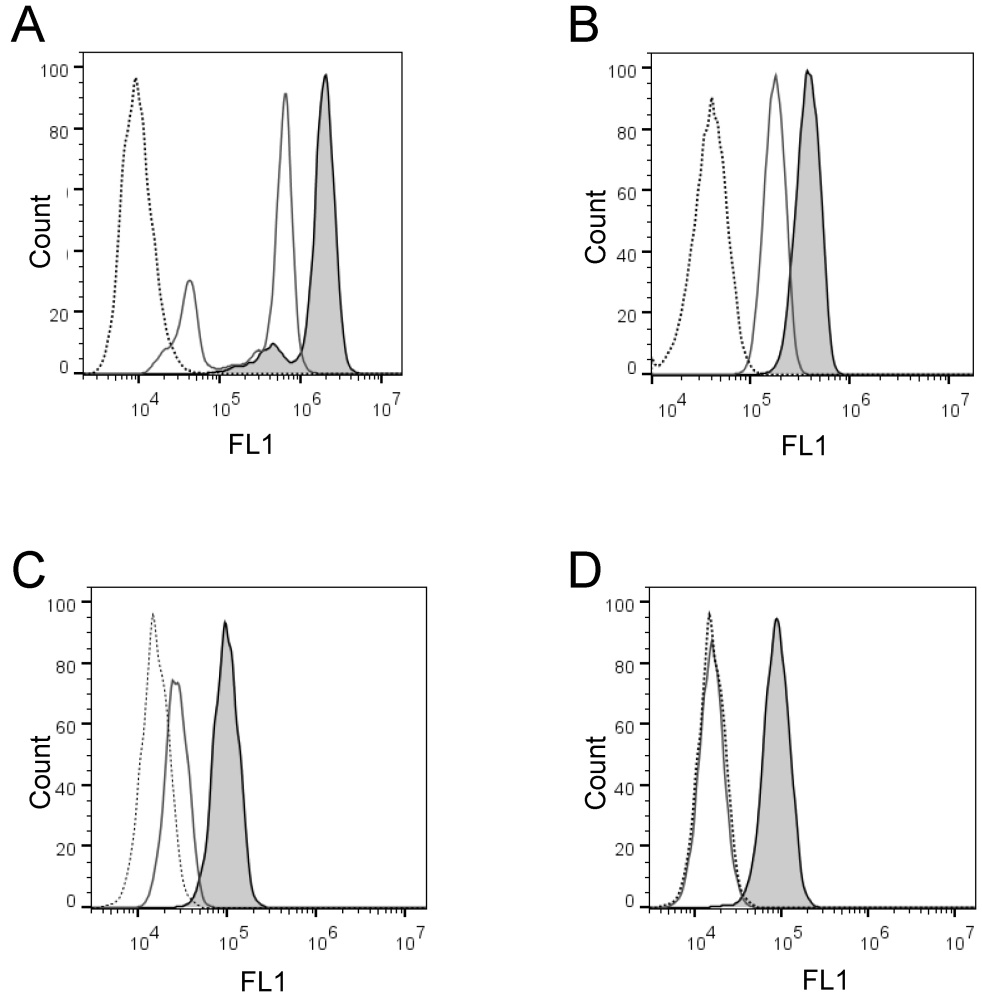
** Activity of cell surface-expressing enzymes can process PEPDAB005 substrate and its fluorescent product can label individual cells that can be measured using flow cytometry. (A, C)** H441 cells were incubated in the absence (dashed line empty histogram) or presence of 10 μM PEPDAB005 at 0^o^C (full line empty histogram) or 21^o^C (full line gray histogram) for 30 min, fixed with 2% PFA, **(A)** kept unwashed or **(C)** were washed with PBS, and examined by flow cytometry. **(B, D)** K562 cells were incubated in the absence (dashed line empty histogram) or presence of 5 μM PEPDAB005 at 0^o^C for 60 min, washed, fixed with 0.005% GAL at 0^o^C for 30 min, incubated for additional 30 min either at 0^o^C (full line empty histogram) or 21^o^C (full line gray histogram), post-fixed with 2% PFA, **(B)** kept unwashed or **(D)** were washed with PBS, and examined by flow cytometry.

Next, we wanted to confirm these findings and quantify the cell-membrane associated enzyme activity on the individual cells in large cell populations. To do that, we developed two flow cytometry enzyme-activity assays using H441 and K562 cells: the live cell assay and the fixed cell assay, respectively. Initially, we detected with both assays distinct increases of the cell-associated fluorescence after cell incubation in the presence of PEPDAB005 at 21^o^C, as compared to the low cell-associated fluorescence after cell incubation in the absence or presence of PEPDAB005 at 0^o^C (Figs. 2A, 2B). Depending on their treatments, individual cells of both cell lines showed different ranges of different fluorescence levels (mean-fluorescence intensity, MFI), being log-normally distributed in characteristic bell-shape histograms. Fluorescence distributions obtained after cell staining with PEPDAB005 at 21^o^C and their superior fluorescence levels to those of the control cells incubated at 0^o^C were very similar to those observed with the same cell lines stained with anti-ADAM10 or isotype-control fluorescent antibodies, respectively (Fig. 2, Supplementary Fig. 1) 22. Importantly, a large portion of the cell-associated fluorescence that was developed in the presence of PEPDAB005 continued to be associated with cells after their extensive washing, especially in the assays performed at 21^o^C and more in the fixed cell assay (20%, live cell assay; 89% fixed cell assay) (Figs. 2C, 2D). These findings suggest that in both assays, but more markedly in the fixed cell assay, the processed PEPDAB005 fluorescent product may specifically bind to reactive enzymes (i.e., ADAM10) on the cell membrane and, thus, could serve as a quantitative marker of the individual cell membrane enzyme activity. They also indicate that the fixed cell assay could better differentiate than the live cell assay the cell-membrane associated enzyme activity at 0^o^C and 21^o^C, especially after cell washing (live cell assay, 2-fold MFI difference; fixed cell assay, 5.5-fold MFI difference), and could be more specific and robust.

### PEPDAB005 substrate specifically bound to cell-membrane enzymes can be crosslinked with glutaraldehyde without affecting enzyme activity and substrate processing ability

**Figure 3:**
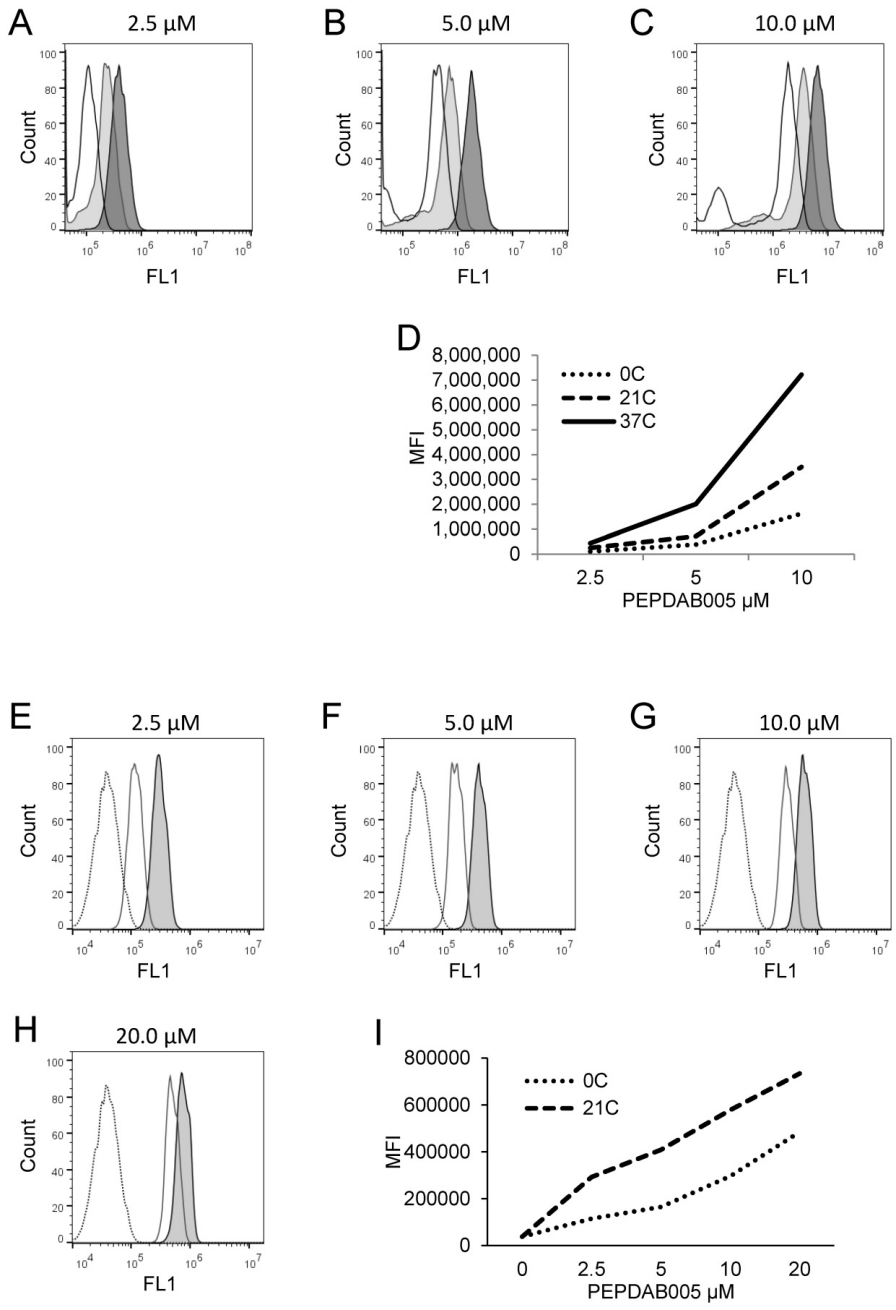
** Cell processing of PEPDAB005 substrate and staining with its fluorescent product depend on substrate concentration and temperature. (A-D)** H441 cells were incubated for 30 min in the presence of **(A)** 2.5 μM, **(B)** 5 μM and **(C)** 10 μM PEPDAB005 at 0^o^C (full line, empty histograms), 21^o^C (full line light gray histograms) and 37^o^C (full line dark gray histograms), fixed with 2% PFA and examined by flow cytometry. **(D)** MFI data of **A-C** figures are summarized. **(E-I)** K562 cells were incubated for 60 min in the absence (dashed line empty histograms) or presence of **(E)** 2.5 μM, **(F)** 5 μM, **(G)** 10 μM and **(H)** 20 μM PEPDAB005 substrate, washed, fixed with 0.005% GAL at 0^o^C for 30 min, incubated for additional 30 min at 0^o^C, (full line empty histograms) or 21^o^C (full line light gray histograms), post-fixed with 2% PFA and analyzed by flow cytometry. **(I)** MFI data of **E-H** figures are summarized.

As GAL could potentially cause nonspecific fluorescence and loss of enzyme activity, we next determined its optimal concentration that could both produce the lowest nonspecific fluorescence and effective crosslinking of the specifically bound substrate to cell-membrane expressing enzymes without affecting the enzyme activity and substrate processing ability (Supplementary Figs. 3A-3E). We found that a relatively wide range of low GAL concentrations (0.0025 to 0.02%) did not inhibit enzyme activity and substrate processing ability (Supplementary Fig. 3E), and that 0.005% GAL supported consistent substrate processing and robust cell staining with the fluorescent product of 2.5 μM and higher PEPDAB005 concentration (Supplementary Fig. 3, Fig. 3). Notably, binding 0.625 μM and 1.25 μM PEPDAB005 to K562 cells, and fixation with 0.005% GAL and concurrent incubation at 21^o^C generated equal levels of low fluorescence, indicating that these concentrations of PEPDAB005 and GAL produced only the nonspecific fluorescence. Therefore, in the fixed cell assay, cells stained with thus low PEPDAB005 concentration in the presence of a low GAL concentration, which produce the same fluorescence as GAL alone, could serve as an optimal negative control to measure the specific fluorescence staining with higher substrate concentrations (Supplementary Fig. 3E, Fig. 3I). Additionally, after the binding of 5 μM PEPDAB005 to cell-membrane expressing enzymes and fixation with 0.0025% or 0.005% GAL, a large portion of the specific fluorescence developed at 21^o^C, but not at 0^o^C, remained associated with cells after their extensive washing (Supplementary Fig. 2), indicating again specific and non-reversible binding of the fluorescent substrate product to reactive cell-membrane associated enzymes.

### PEPDAB005 substrate is processed by and labels cells in temperature-, substrate-concentration-, and time-dependent manners

**Figure 4:**
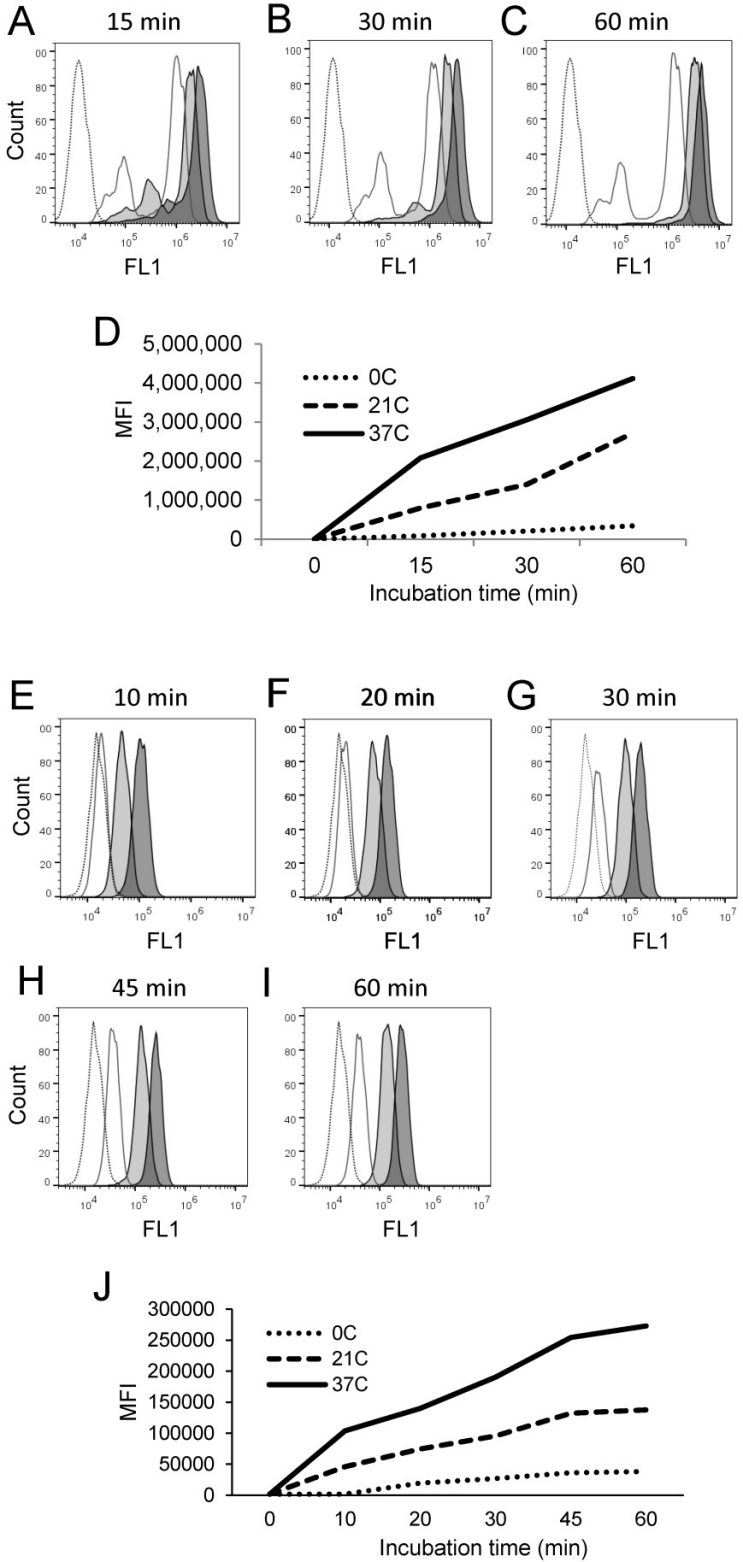
** Cell processing of PEPDAB005 substrate and staining with its fluorescent product depend on time and temperature. (A-D)** H441 cells were incubated in the absence (dashed line empty histograms) or presence of 10 μM PEPDAB005 for **(A)** 15 min, **(B)** 30 min or **(C)** 60 min at 0^o^C (full line empty histograms), 21^o^C (full line light gray histograms) and 37^o^C (full line dark gray histograms), fixed with 2% PFA and examined by flow cytometry. **(D)** MFI data of **A-C** figures are summarized. **(E-J)** K562 cells were incubated for 60 min in the absence (dashed line empty histogram) or presence of 5 μM PEPDAB005 for 60 min, washed, fixed with 0.005% GAL at 0^o^C for 30 min, incubated for **(E)** 10 min, **(F)** 20 min, **(G)** 30 min, **(H)** 45 min or **(I)** 60 min at 0^o^C (full line empty histograms), 21^o^C (full line light gray histograms) or 37^o^C (full line dark gray histograms), post-fixed with 2% PFA and analyzed by flow cytometry. **(J)** MFI data of **E- I** figures are summarized.

Canonical enzymatic reactions are dependent on temperature, substrate-concentration and time of incubation. We tested whether the enzymatic reactions measured with the live cell assay and the fixed cell assays have these characteristics (Figs. 3, 4). We obtained similar results with both assays. Relative to the cells incubated in the absence of substrate displaying unchangeable low fluorescence levels, those incubated in the presence of PEPDAB005 showed progressive increases of fluorescence with the increases of incubation temperature from 0^o^C to 21^o^C and, additionally, from 21^o^C to 37^o^C, reflecting the temperature-dependent rises of processed substrate and enzymatic activity levels (live cell assay, Figs. 3A-3D, 4A-4D; fixed cell assay, Figs. 3E-3I, 4E-4J). The specific fluorescence was detectable with 2.5 μM PEPDAB005 and continuously increased with the augmentation of substrate concentration, reaching its maximal level with 10 μM (live cell assay, Figs. 3A-3D; fixed cell assay, Supplementary Figs. 3A-3E, Figs. 3E-3I). In addition, the enzyme-activity associated specific fluorescence continuously increased with the time of incubation (live cell assay, Figs. 4A-4D, Supplementary Fig. 4B; fixed cell assay, Figs. 4E-4J).

The differences of specific fluorescence developed in the presence of PEPDAB005 at 0^o^C, 21^o^C and 37^o^C were again greater using the fixed cell assay than the live cell assay. In addition, after cell co-incubation with 10 μM PEPDAB005 at different temperatures and for different time periods, increasing fractions of generated fluorescence remained associated with cells after their extensive washing, further indicating temperature- and time-dependent binding of the fluorescent PEPDAB005 product to cell-membrane associated reactive enzymes (Supplementary Figs. 4A-4B). These findings demonstrate that both assays can measure cell-membrane associated enzyme activity at the single cell level and indicate that the fixed cell assay might be superior.

### The assays measure lower levels of enzyme activity on ADAM10 knockout than wild-type cells

**Figure 5:**
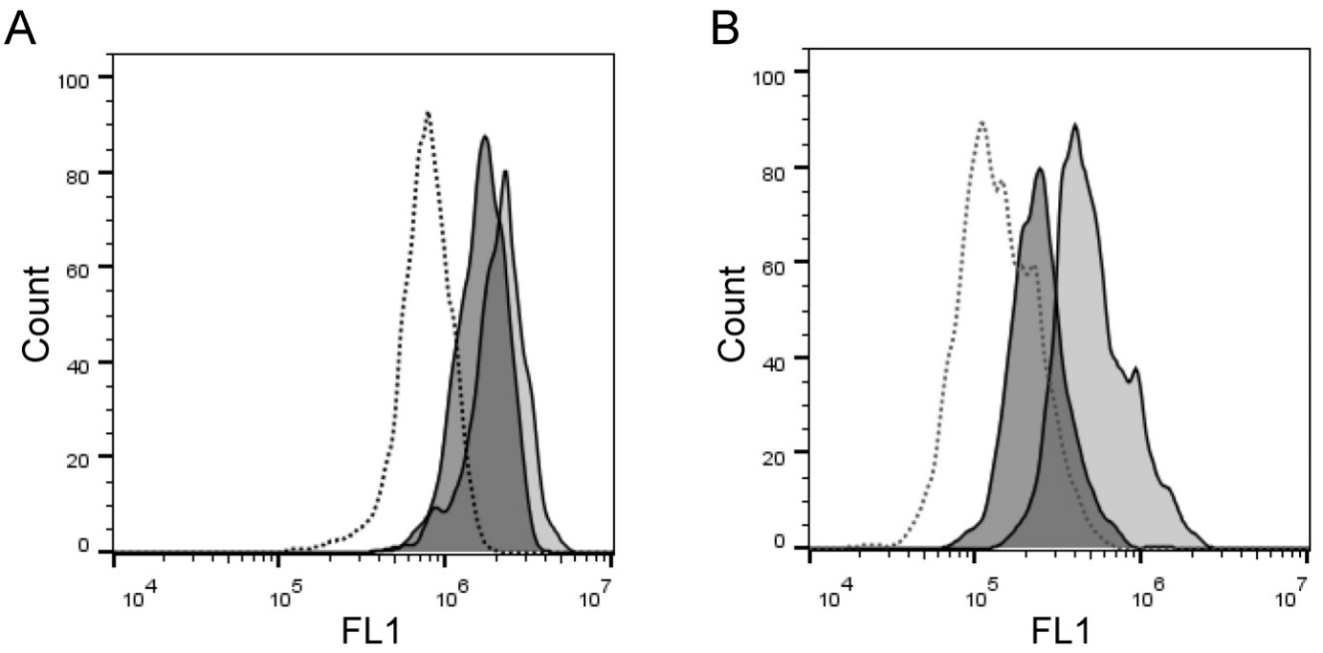
** Cell processing of PEPDAB005 substrate and staining with its fluorescent product detect decreased enzymatic activity of ADAM10^-/-^ MEFs relative to ADAM10^+/-^ MEFs. (A)** ADAM10^+/-^ and ADAM10^-/-^ MEFs were incubated for 30 min at 21^o^C in the absence (dashed line empty histogram) or presence of 10 μM PEPDAB005 substrate (ADAM10^+/-^ MEFs, full line light gray histogram; ADAM10^-/-^ MEFs, full line dark gray histogram), fixed with 2% PFA and examined by flow cytometry. **(B)** Cells were incubated for 60 min at 0^o^C in the absence (dashed line empty histogram) or presence of 5 μM PEPDAB005 substrate (ADAM10^+/-^ MEFs, full line light gray histogram; ADAM10^-/-^ MEFs, full line dark gray histogram), washed, fixed with 0.005% GAL at 0^o^C for 30 min, incubated for additional 30 min at 21^o^C^,^ post-fixed with 2% PFA and analyzed by flow cytometry.

To assess the ability of the assays to measure specific cell-membrane associated enzyme activities, we examined whether the assays could detect and quantify presence and absence of ADAM10 sheddase activity on ADAM10^+/-^ and ADAM10^-/-^ MEFs, respectively. We found with both assays lower staining levels with PEPDAB005, or lower enzyme activity of ADAM10^-/-^ MEFs than ADAM10^+/-^ MEFs (Fig. 5). However, the fixed cell assay was superior at distinguishing the absence and presence of ADAM10 enzyme activity (live cell assay, 1.25-fold MFI difference; fixed cell assay, 3.3-fold MFI difference).

### The assays measure different levels of enzyme activity on lymphocytes and monocytes expressing different levels of ADAM10

**Figure 6:**
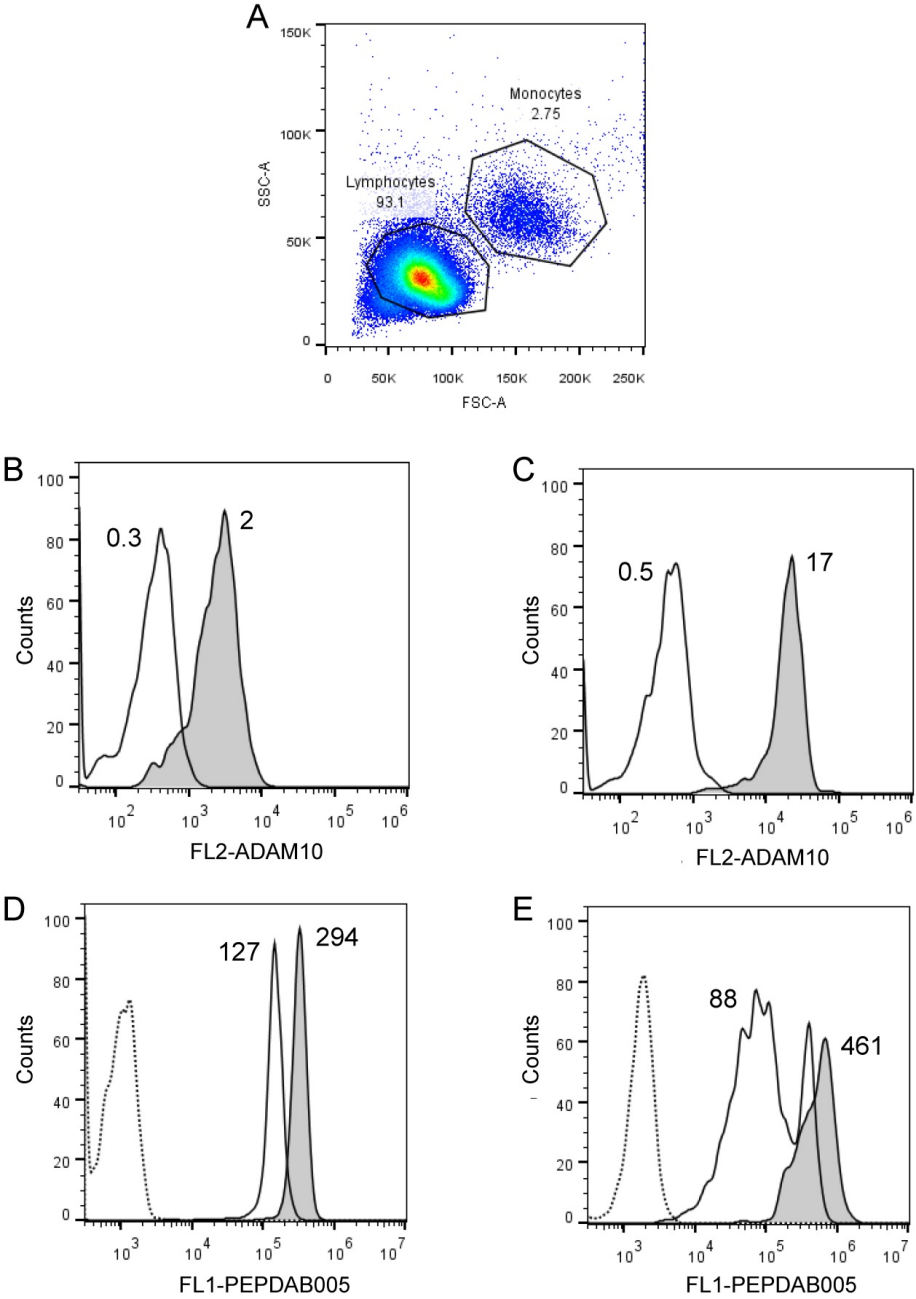
** Cell processing of PEPDAB005 substrate and staining with its fluorescent product detect different enzymatic activities on lymphocytes and monocytes.** Healthy human PBLs **(A)** were isolated using Ficoll-Hypaque gradient centrifugation, stained with 10 µg of PE-conjugated isotype-control (dashed line empty histograms) and anti-human ADAM10 mAbs (full line empty histograms) **(B, C)**, or incubate for 30 min without (dashed line empty histograms) or with 10 µM PEPDAB005 at 0^o^C (full line empty histograms) and 21^o^C (full line grey histograms), fixed with PFA and analyzed by flow cytometry **(D, E)**. **(A)** SSC and FSC dot-plot of PBLs and gating of lymphocytes and monocytes, **(B)** ADAM10 expression on lymphocytes, **(C)** ADAM10 expression on monocytes, and staining of **(D)** lymphocytes and **(E)** monocytes with PEPDAB005 fluorescent product are shown. Numbers in figure** (A)** are % of cells. Numbers in figures **(B)** and **(C)** are MFI (X 10^3^). Numbers in figures **(D)** and **(E)** are MFI (x 10^3^) after subtraction of auto fluorescence MFI (dashed line empty histograms).

We frequently observed within cell lines a high heterogeneity of cells in their staining levels with PEPDAB005 fluorescent product (Figs. 2A, 3A-3C, 4A-4C). These findings indicate that even relatively homogenous cell populations such as cell lines may contain cells having various levels of cell-membrane associated enzyme activity. We next examined whether the cell-membrane enzyme activity assays could measure different levels of enzyme activity and, thus, discriminate phenotypically and functionally distinct cells such as lymphocytes and monocytes. We performed this examination using freshly isolated unfractionated population of human PBL. Indeed, we found that monocytes expressed on their cell surface 5.1-fold higher levels of ADAM10 (Figs. 6A, 6B) and developed at 21^o^C (relative to 0^o^C) 2.3-fold higher levels of cell-associated PEPDAB005 fluorescent product than lymphocytes (Fig. 6C, 6D)**.** These findings demonstrate that the enzyme activity assays can accurately distinguish cells displaying different cell-membrane associated enzyme activities in heterogeneous cell populations.

### Co-staining of cell-surface ADAM10 and cell-membrane enzyme activity

**Figure 7:**
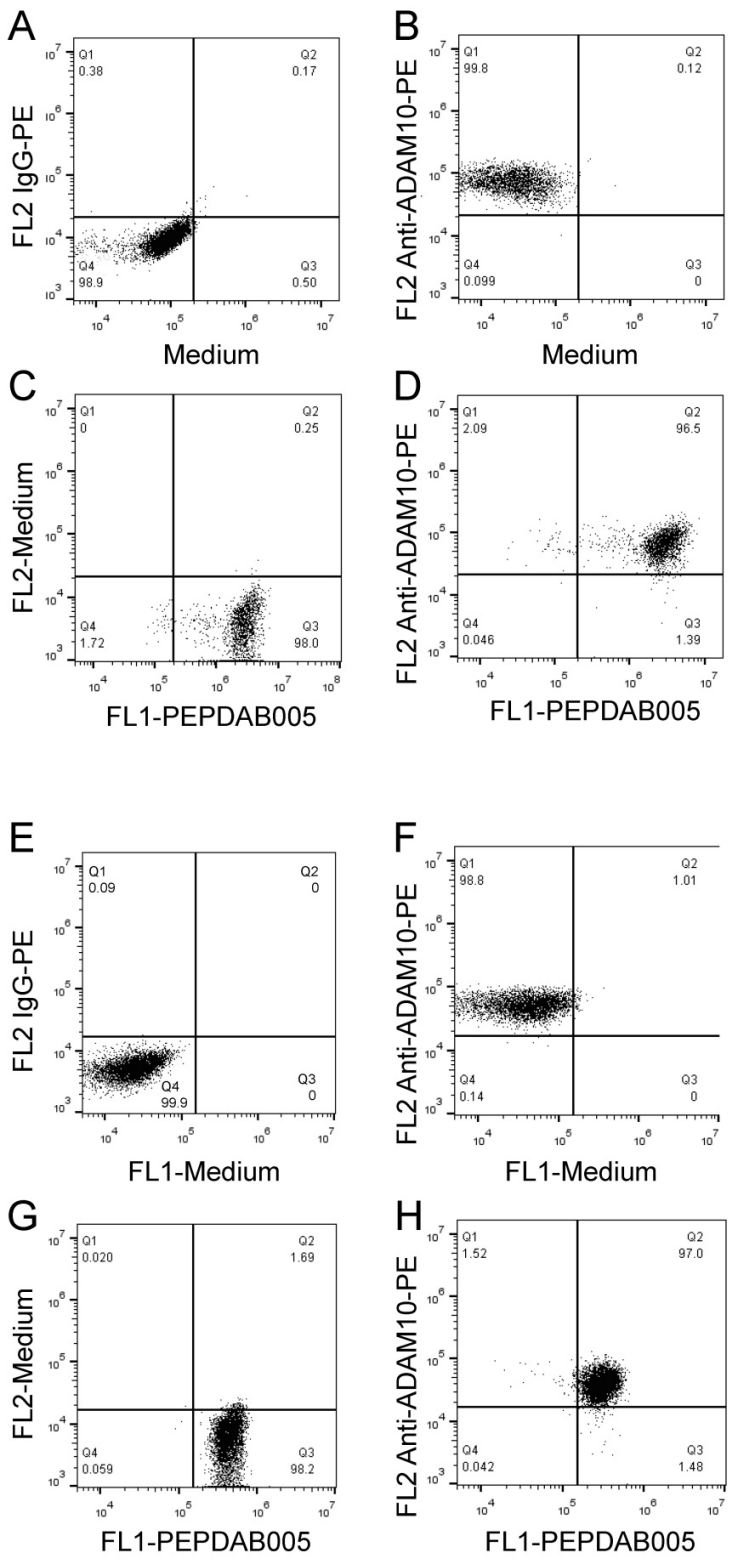
** PEPDAB005 processing and enzyme specific antibody staining can be combined to co-label enzyme activity and particular enzyme molecule on individual-cell surface.** H441 cells were stained with **(A)** 10 µg PE-conjugated IgG control antibody or **(B)** PE-conjugated anti-human ADAM10 antibody,** (C)** incubated in the presence of 10 μM PEPDAB005 at 21^o^C for 30 min; and **(D)** stained first with 10 µg PE-conjugated anti-human ADAM10 antibody, washed and incubated in the presence of 10 μM PEPDAB005 at 21^o^C for 30 min. After these incubations, cells were fixed with 2% PFA. K562 cells were stained with **(E)** 10 µg PE-conjugated IgG control antibody or **(F)** PE-conjugated anti-human ADAM10 antibody; **(G)** incubated in the presence of 5 μM PEPDAB005 at 0^o^C for 60 min, washed, fixed with 0.005% GAL at 0^o^C for 30 min, and incubated at 21^o^C for 30 min; and **(H)** co-incubated with 10 µg PE-conjugated anti-human ADAM10 antibody and 5 μM PEPDAB005 at 0^o^C for 60 min, washed, fixed with 0.005% GAL at 0^o^C for 30 min, incubated at 21^o^C for 30 min, and post-fixed with 2% PFA. Cells were analyzed using two-color flow cytometry.

Our published (26) and above presented findings suggest that PEPDAB005 processing could measure cell-membrane associated ADAM10 sheddase activity on cells expressing high ADAM10 and low ADAM17 levels. To more directly examine this possibility, two-color cell-surface flow cytometry assays were developed by combining the antibody immunostaining of ADAM10 and enzyme activity staining using the live cell assay (Fig. 7A-7D) and the fixed cell assay (Fig. 7E-7H). Using both assays, we clearly co-stained individual H441 and K562 cells with anti-ADAM10 antibody and PEPDAB005 fluorescent product (Figs 7D, 7H, respectively). Importantly, the levels of ADAM10 and enzyme-activity staining closely correlated on each individual cell. These findings show the association of cell-surface expressed ADAM10 and cell-membrane associated enzyme activity at the single cell level and, in conjunction with published (22, 26) and here presented data (Supplementary Fig. 1, Figs. 5, 6), indicated that PEPDAB005 processing assays mostly measure ADAM10 sheddase activity on H441 and K562 cells.

## Discussion

To accurately investigate biology of the cell-membrane expressing enzymes, methods to measure catalytic activities on the cell surface of individual cells are required, but unavailable. According to the current notion, the enzymatic processing of substrate ends by a rapid dissociation of the enzyme-substrate product complex, allowing an enzyme rapid recycling and recurring processing of multiple substrate molecules 24. The potential rapid detachment of processed substrates from plasma membrane enzymes could be a major obstacle for developing single-cell membrane associated enzyme activity assays. However, the enzymatic processing of FRET substrates leads to their reversible conformational changes, but not to irreversible canonical cleavage 15, and the enzyme activity-generated FRET substrate product could retain the ability of unprocessed substrate to bind to reactive enzymes and, thus, to label the cell surface. Here we tested and provided support for this possibility by confocal microscopy imaging and two newly developed flow cytometry-based assays that measured individual-cell membrane associated enzyme activity using PEPDAB005 FRET substrate. The novel flow cytometry assays are relatively simple, easy to perform, robust, precise, high content and high throughput, being able to measure different levels of individual-cell membrane enzyme activity of both homogenous and heterogeneous large cell populations. In addition, the assays can be combined with conventional specific immunostaining of plasma membrane enzymes, cell phenotype and functional markers, to directly associate cell-membrane proteolytic activities with specific cell-membrane expressing enzymes, cell types and their functions.

One of the assays exploits the newly discovered ability of the naturally processed PEPDAB005 substrate to continue binding to cell-membrane expressing enzymes during an enzymatic reaction on the cell surface. The other assay is based on the novel observation that preformed specific complex of PEPDAB005 substrate and cell-membrane expressing enzymes can be mildly crosslinked with low GAL concentration in order to retain unaffected both enzyme activity and substrate-processing ability, and to generate a fluorescent substrate product crosslinked to the individual-cell membrane expressing reactive enzymes. Both methods produced a cell-membrane bound substrate product that can be permanently fixed with PFA, to enable a prolonged cell storage without loss of the fluorescent substrate product. In both assays, the processed substrate consistently labeled cell membrane of single cells. Substrate product remained largely cell bound after cell washing, and the assays typically measured individual-cell membrane associated enzyme activity in the substrate-concentration-, incubation-time- and temperature-dependent fashion. However, the specific fluorescence was greater and the differences between fluorescence levels of cells stained with PEPDAB005 at 0^o^C and 21^o^C were larger using the fixed cell assay than the live cell assay. In addition, the fixed cell assay measured larger differences between enzyme activities of ADAM10 wild-type and ADAM10 deficient MEFs. Thus, the fixed cell assay is more sensitive than the live cell assay.

A major dilemma of this study, especially when performed with the live cell assay, was whether the fluorescent product generated during specific enzymatic reactions was bound and remained bound to the corresponding cell-membrane expressing reactive enzymes and, thus, selectively labeled surface of single cells where the enzymatic reaction occurred, or was released from the reactive enzymes, diffused and non-selectively labeled cells. If the latter possibility would be correct, all cells within a tested cell population, no matter whether they were homogenous (i.e., cell lines) or heterogeneous (i.e., peripheral blood mononuclear cells), would have similar MFI, and the fluorescent substrate products generated by cell-membrane associated enzyme activity could be washed out from the cell surface. Contrarily, cells stained with the enzyme-activity generated fluorescent substrate product showed a range of different individual-cell fluorescence levels that were distributed in approximately log-normal histograms. Importantly, even within homogenous cell populations such as cell lines, besides the main fluorescence intensity histogram, an additional smaller histogram and/or a tail of lower fluorescence intensity were observed, indicating different enzyme activities and probably functional states of individual cells within these cell populations. More compellingly, within a heterogeneous cell population of human peripheral blood mononuclear leukocytes, the cell-membrane enzyme activity assays clearly measured quantitatively different enzyme activities on the phenotypically and functionally distinct lymphocytes and monocytes expressing different levels of ADAM10. Most importantly, the extensive washing of cells did not remove a large proportion of the enzyme-activity generated cell-membrane bound fluorescent substrate product detected by the either confocal microscopy or novel flow cytometry methods. Taken together, these findings suggest that the processed PEPDAB005 substrate specifically binds and remains bound to cell-membrane expressed proteolytic enzymes and thus fluorescently labels individual cells.

The assays detected a decrease of enzyme activity on ADAM10 knockout cells, measured enzyme activity that correlated with different ADAM10 expression on lymphocytes and monocytes, and detected co-staining of enzyme activity and anti-ADAM10 antibody on individual cells. H441 22 and K562 (present study) cells showed high ADAM10 and low ADAM17 expression levels on the cell surface and mediated high ADAM10 and low ADAM17 sheddase activity 22, 26. In addition, enzymatic processing of PEPDAB005 has demonstrated features of a surrogate marker of ADAM10 sheddase activity for the cells and tissues expressing high ADAM10 and low ADAM17 levels 26. Therefore, the new assays performed with PEPDAB005 substrates preferentially measured ADAM10 sheddase activity on the cell membrane of these two cell lines expressing high ADAM10 and low ADAM17 levels.

In conclusion, we newly developed and defined two single-cell membrane-associated enzyme activity assays, and provided indications on their potential applications to study plasma-membrane enzyme biology related to particular cell types and cell functions (Table 1). The cells to be studied could be freshly isolated or cultured, homogenous or heterogeneous, immature or mature, normal or transformed, unstimulated or stimulated. Importantly, ADAM10 and/or ADAM17 sheddase activities have been shown to be highly upregulated on cancer cells, in cancer tissues and on circulating exosomes of cancer patients, and have demonstrated a considerable biomarker potentials 22, 26, 27. Therefore, the novel assays could be also implemented in the clinic to directly evaluate diagnostic and prognostic abilities of the individual-cell membrane associated enzyme activity of the blood- and tissue-resident cancer cells isolated from cancer patients.

## Supplementary Material

Supplementary figures and tables.

## Figures and Tables

**Table 1:** New observations, approaches and findings in the present study.

New observations:	
	(a) PEPDAB005 FRET substrate can be processed by live-cell enzymes to generate a fluorescent product that binds to and labels individual-cell membrane.
	(b) PEPDAB005 substrate can be sequentially bound and glutaraldehyde crosslinked to plasma membrane enzymes without substantially affecting enzymatic activity, substrate processing and generation of an enzyme-coupled fluorescent substrate product that labels individual cells.
New approaches (assays):	
	(a) Live-cell membrane associated enzyme activity individual-cell assay.
	(b) Fixed-cell membrane associated enzyme activity individual-cell assay.
New results:	
	(a) Confocal microscopy demonstrates co-localization on individual cells of cell-membrane specific staining with DiD and enzyme activity staining with processed PEPDAB005 substrate product.
	(b) The novel assays measure specific increases of cell-associated PEPDAB005 processing product in substrate-concentration-, temperature- and time-dependent manners.
	(c) The novel assays measure different levels of enzyme activity on different cells.
	(d) The novel assays detect the presence, absence and different levels of ADAM10 sheddase activity on different cells.
	(e) The novel assays can be combined with cell-type, cell-function and cell-membrane enzyme specific markers.

## Abbreviations

FPS: Fluorogenic peptide substrate
FRET: Fluorescence-resonance energy transfer
MFI: Mean-fluorescence intensity
ADAM: A disintegrin and metalloproteinase
Live cell assay: Live cell/natural substrate-processing assay
Fixed cell assay: Fixed cell/crosslinked substrate-enzyme complex processing assay
