# Supplementary material for: Development of flow cytometry assays for measuring cell-membrane enzyme activity on individual cells
Source: J Cancer. 2020 Jan 1;11(3):702–15. doi: 10.7150/jca.30813 (PMC6959049; doi:10.7150/jca.30813)
Supplement: Supplementary file 1 — Supplementary figures and tables. [file jcav11p0702s1.pdf]

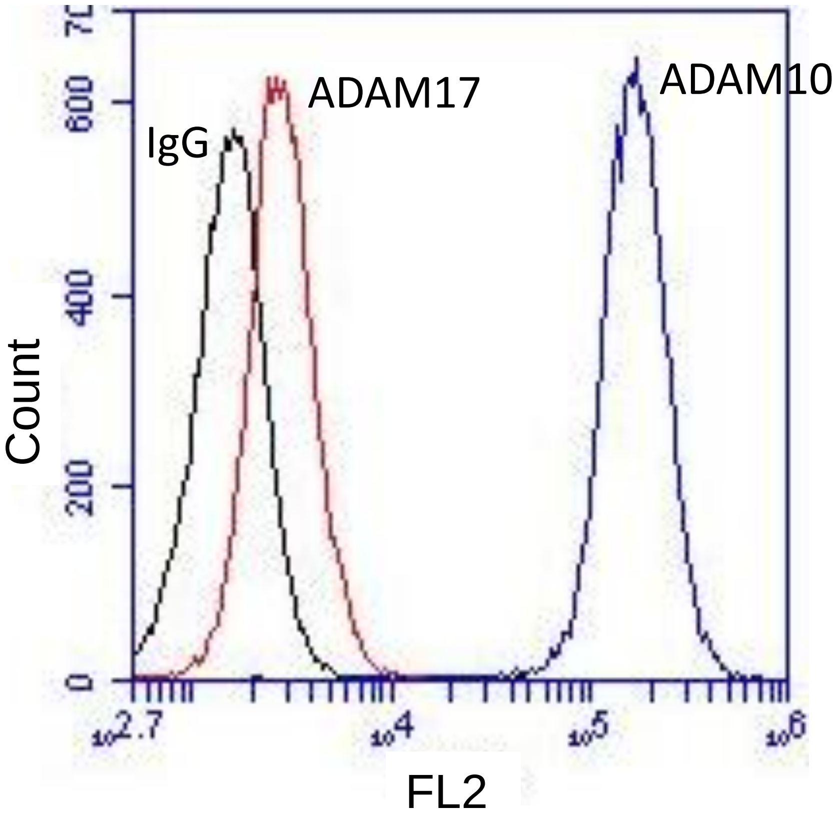

Supplemental Figure 1. *K562 cells express on plasma membrane high ADAM10 and low ADAM17 levels.* K562 cells were stained with PE-conjugated isotype control, anti-human ADAM10 and anti-human ADAM17 monoclonal antibodies, and examined by single-color flow cytometry. Presented data are histograms of fluorescence intensity.

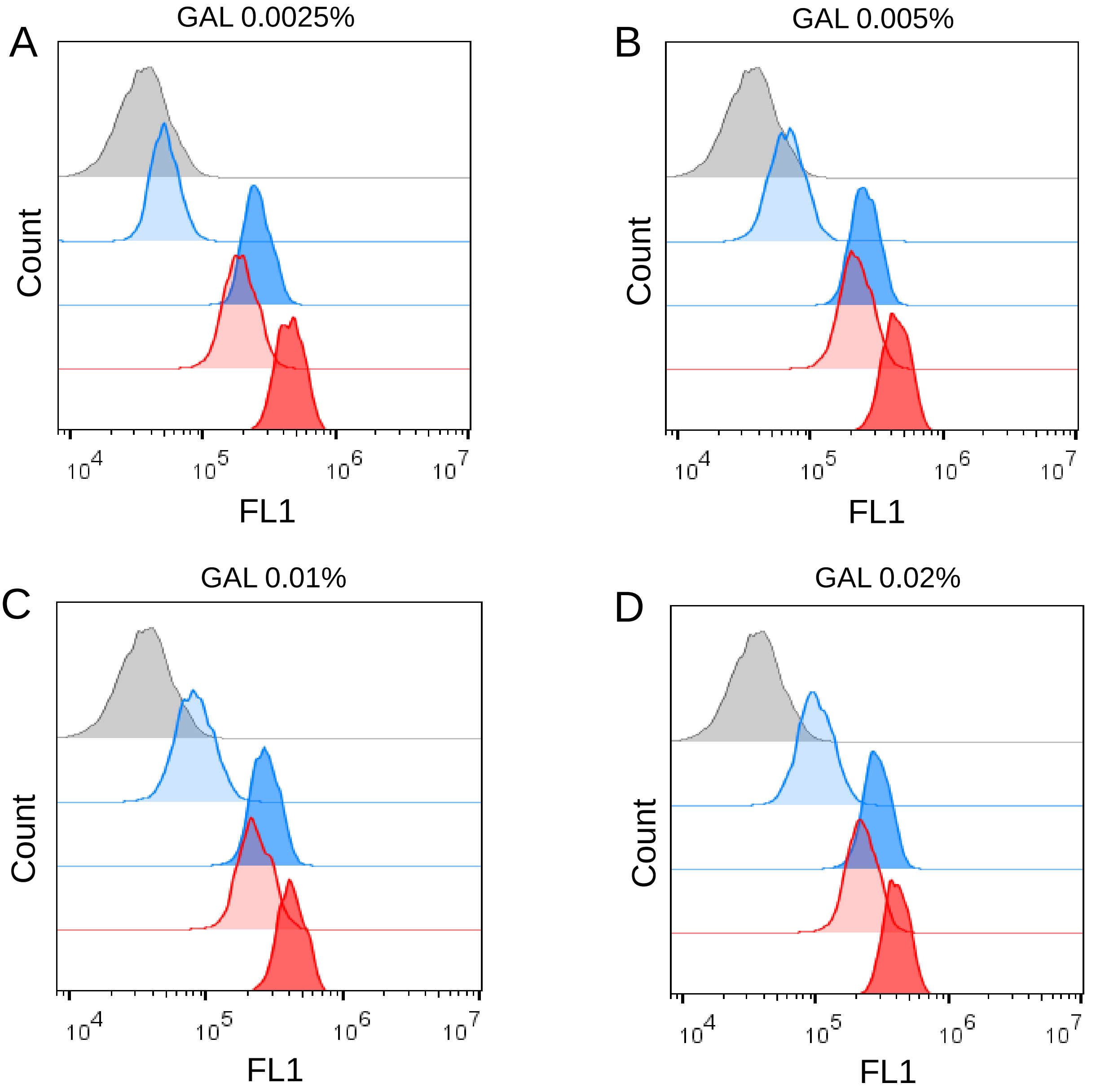

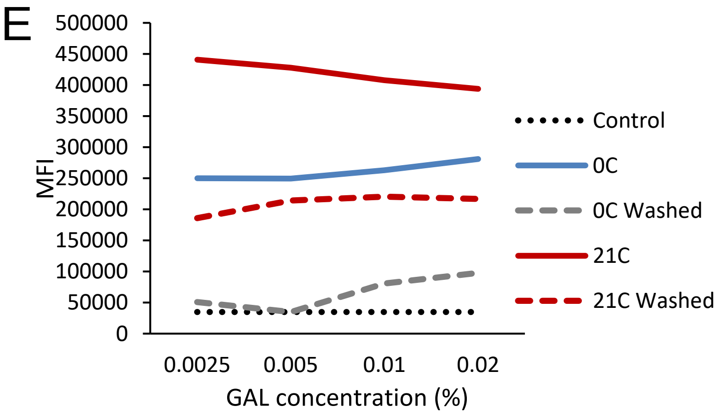

Supplemental Figure 2. *Low concentrations of GAL support PEPDAB005 processing and fix the processed fluorescent substrate to cell surface.* K562 cells were incubated in the absence (grey histograms) and presence of 5 µM PEPDAB005 at 0°C for 60 min, washed, incubated in the presence of 0.0025% ***A***, 0.005% ***B***, 0.01% ***C*** and 0.02% ***D*** GAL at 0°C for 30 min, continued to incubate at 0°C (blue histograms) or 21°C (red histograms) for additional 30 min, post-fixed with 2% PFA, kept unwashed (dark-color histograms) or were washed before FACS analysis (light-color histograms). ***E*** MFI data presented in ***A***-***D*** figures are summarized.

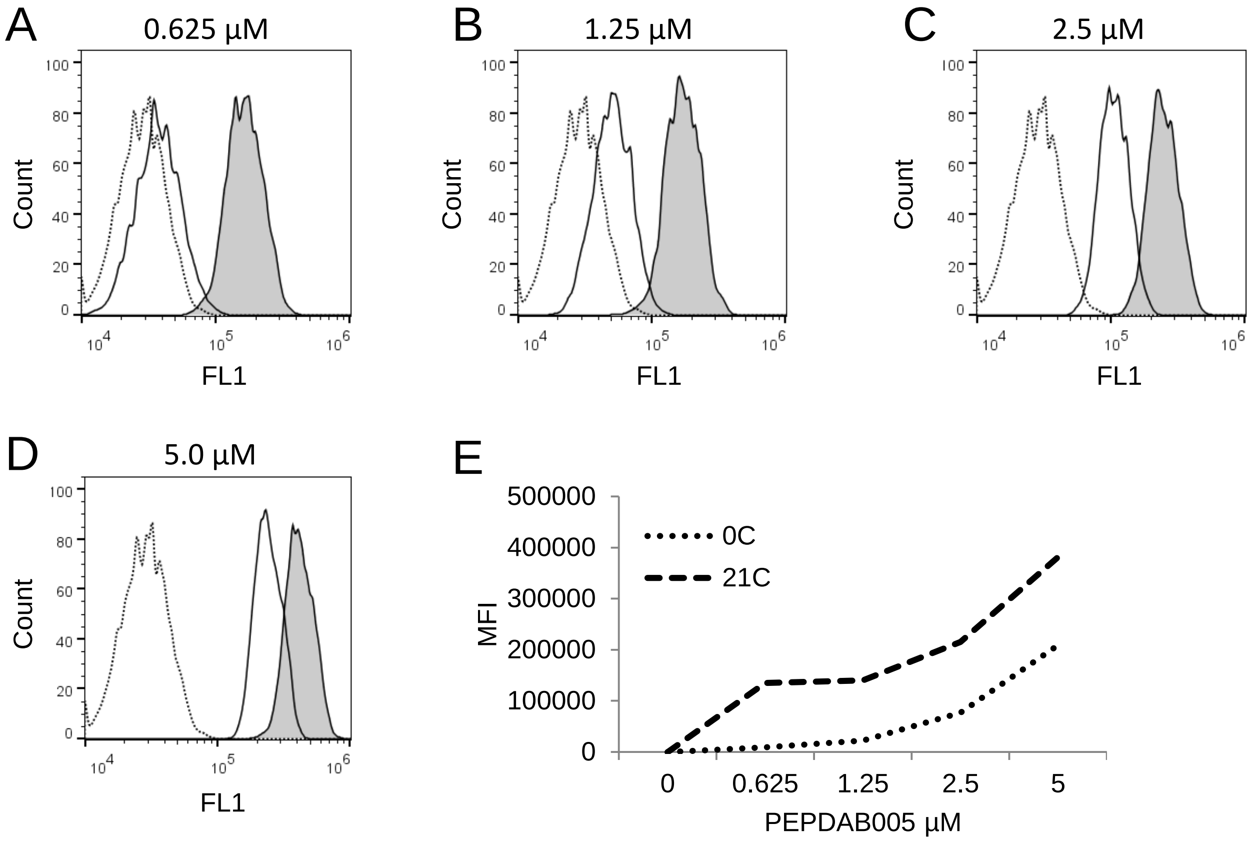

Supplemental Figure 3. *In the presence of GAL, processed PEPDAB005 substrate can be detected on the cell surface using at least 2.5 µM PEPDAB005.* K562 cells were incubated in the absence (dashed line empty histograms) or presence of 0.625 µM ***(A)***, 1.25 µM ***(B)***, 2.5 µM ***(C)*** and 5.0 µM PEPDAB005 substrate ***(D)*** at 0°C for 60 min, washed, fixed with 0.005% GAL at 0°C for 30 min, incubated at 0°C (full line empty histograms) or 21°C (full line gray histograms) for 30 min, post-fixed with 2% PFA and analyzed by FACS. ***(E)*** MFI data presented in ***A-D*** figures are summarized.

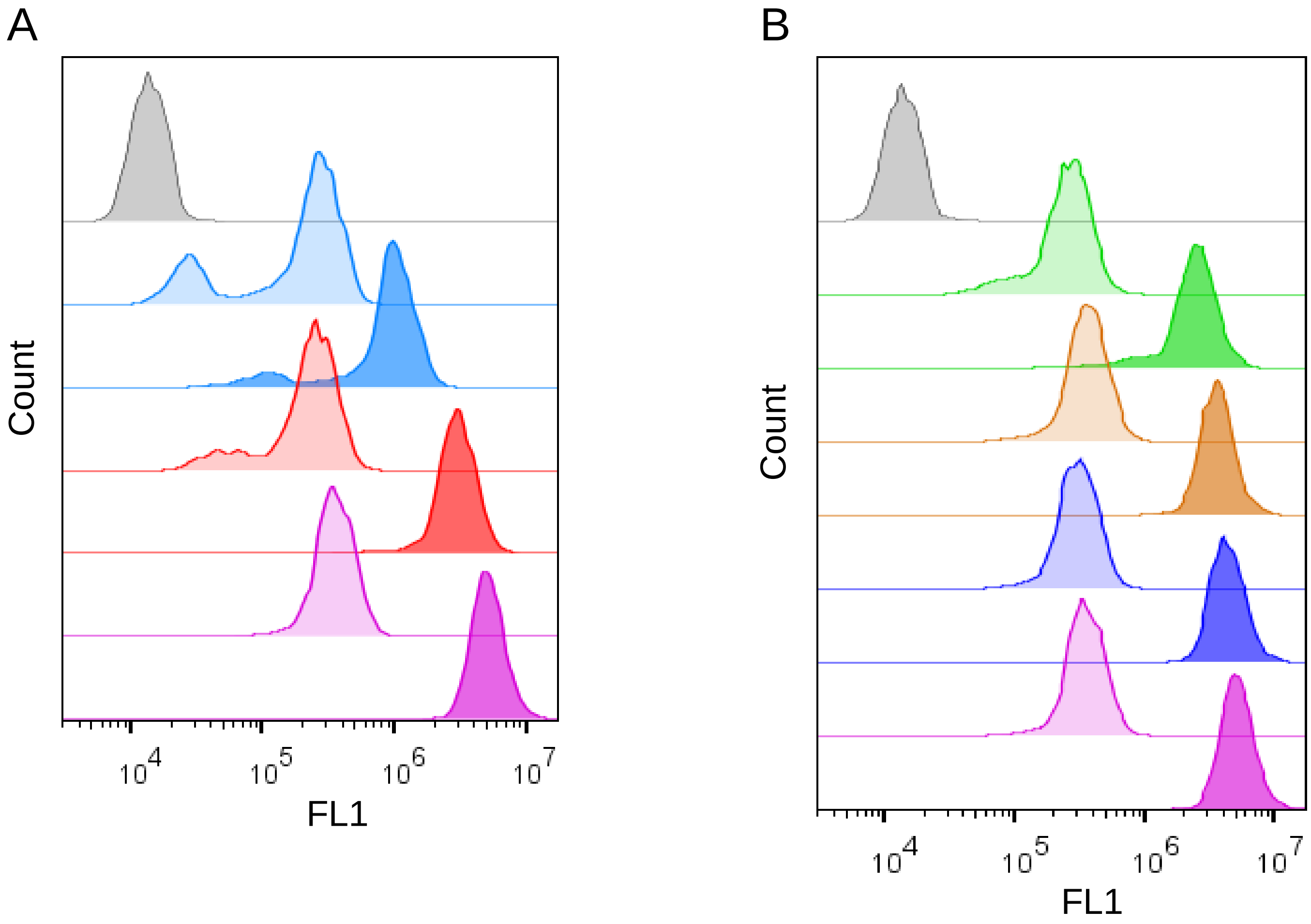

Supplemental Figure 4. *A significant portion of cell-associated processed PEPDAB005 substrate cannot be washed out and is dependent on temperature and incubation time.* ***(<u>A</u>)*** H441 cells were incubated in the absence (grey histogram) or presence of 10 µM PEPDAB005 at 0°C (blue histograms), 21°C (red histograms) and 37°C (purple histograms), fixed with 2% PFA, left unwashed (dark color histograms) or were washed (light color histograms), and analyzed by FACS. ***(<u>B</u>)*** H441 cells were incubated in the absence (grey histogram) or presence of 10 µM PEPDAB005 at 37°C for 15 min (green histograms), 30 min (orange histograms), 45 min (blue histograms) and 60 min (purple histograms), fixed with 2% PFA, left unwashed (dark color histograms) or were washed (light color histograms), and analyzed by FACS.
